# Supplementary material for: Machine Learning Accelerates Crystallization for Structure Determination
Source: Angew Chem Int Ed Engl. 2026 May 4;65(25):e1218503. doi: 10.1002/anie.1218503 (PMC13266958; doi:10.1002/anie.1218503)

# checkCIF/PLATON report

Structure factors have been supplied for datablock(s) lcz078

THIS REPORT IS FOR GUIDANCE ONLY. IF USED AS PART OF A REVIEW PROCEDURE FOR PUBLICATION, IT SHOULD NOT REPLACE THE EXPERTISE OF AN EXPERIENCED CRYSTALLOGRAPHIC REFEREE.

No syntax errors found. CIF dictionary Interpreting this report

# Datablock: lcz078

---

```
Bond precision:   C-C = 0.0086 A          Wavelength=1.54184

Cell:            a=8.7193(2)       b=10.7952(2)      c=21.1971(4)
                 alpha=88.455(2)   beta=86.642(2)    gamma=88.502(2)
Temperature:     100 K
```

| | Calculated | Reported |
|---|---|---|
| Volume | 1990.46(7) | 1990.46(7) |
| Space group | P -1 | P -1 |
| Hall group | -P 1 | -P 1 |
| Moiety formula | C15 H3 Ag3 F18 N6, 2(C10 H9 N O) | C15 H3 Ag3 F18 N6, 2(C10 H9 N O) |
| Sum formula | C35 H21 Ag3 F18 N8 O2 | C35 H21 Ag3 F18 N8 O2 |
| Mr | 1251.21 | 1251.21 |
| Dx,g cm-3 | 2.088 | 2.088 |
| Z | 2 | 2 |
| Mu (mm-1) | 12.933 | 12.933 |
| F000 | 1212.0 | 1212.0 |
| F000’ | 1217.52 | |
| h,k,lmax | 11,13,26 | 11,13,26 |
| Nref | 8548 | 8191 |
| Tmin,Tmax | 0.189,0.164 | 0.684,1.000 |
| Tmin’ | 0.084 | |

```
Correction method= # Reported T Limits: Tmin=0.684 Tmax=1.000
AbsCorr = MULTI-SCAN
```

Data completeness= 0.958 Theta(max)= 78.259

R(reflections)= 0.0448( 7013) wR2(reflections)= 0.1147( 8191)

S = 1.074 Npar= 597

---

The following ALERTS were generated. Each ALERT has the format
**test-name_ALERT_alert-type_alert-level.**
Click on the hyperlinks for more details of the test.

---

## Alert level C

```
PLAT213_ALERT_2_C Atom F00U            has ADP max/min Ratio .....        3.1 prolat
PLAT213_ALERT_2_C Atom F011            has ADP max/min Ratio .....        3.1 prolat
PLAT342_ALERT_3_C Low Bond Precision on  C-C Bonds ..............    0.00856 Ang.
PLAT906_ALERT_3_C Large K Value in the Analysis of Variance ......      4.711 Check
PLAT911_ALERT_3_C Missing FCF Refl Between Thmin & STh/L=    0.600         48 Report
                1  1  0,  -4 10  0,  -3 11  0,  -2 12  0,  -6 -8  1,   9 -6  1,
                5 -4  1,   1 12  1,  -6 -8  2,   0  0  2,   2  2  2,  -6 -8  3,
                9  4  6,   4 -2  7,   4 -2  8,   4-10 10,   0 12 10,   1 12 10,
                4 -9 11,   8 -6 11,   3-10 12,   4 -9 12,   4 -8 12,   4 -7 12,
                4 -9 15,   3 -9 16,   3 -8 16,   4 -8 16,   4 -7 16,   3 -8 17,
                   (   18 More Missing: see the .ckf listing file)
PLAT934_ALERT_3_C Number of (Iobs-Icalc)/Sigma(W) > 10 Outliers ..          1 Check
               -2 12  1,
```

---

## Alert level G

```
PLAT007_ALERT_5_G Number of Unrefined Donor-H Atoms .............          2 Report
              H00R  H00S
PLAT083_ALERT_2_G SHELXL Second Parameter in WGHT  Unusually Large      12.56 Why ?
PLAT154_ALERT_1_G The s.u.'s on the Cell Angles are Equal ..(Note)      0.002 Degree
PLAT242_ALERT_2_G Low    'MainMol' Ueq as Compared to Neighbors of       C00W Check
PLAT242_ALERT_2_G Low    'MainMol' Ueq as Compared to Neighbors of       C01C Check
PLAT242_ALERT_2_G Low    'MainMol' Ueq as Compared to Neighbors of       C01D Check
PLAT242_ALERT_2_G Low    'MainMol' Ueq as Compared to Neighbors of       C01Q Check
PLAT242_ALERT_2_G Low    'MainMol' Ueq as Compared to Neighbors of       C01T Check
PLAT432_ALERT_2_G Short Inter X...Y Contact  F00A     ..C00Z     .       2.95 Ang.
                                                 -1+x,y,z  =      1_455 Check
PLAT720_ALERT_4_G Number of Unusual/Non-Standard Labels ..........         87 Note
              Ag01    Ag02    Ag03    F004    F005    F006    F007    F008
              F009    F00A    F00B    F00C    F00D    O00E    F00F    F00G
              F00H    N00I    O00J    N00K    F00L    F00M    N00N    N00O
              N00P    N00Q    N00R    H00R    N00S    H00S    F00T    F00U
              C00V    H00V    C00W    C00X    C00Y    C00Z    C010    H010
              F011    C012    C013    C014    C015    H01A    H01B    H01C
              C016    H016    C017    H017    C018    C019    H019    C01A
              H01D    C01B    C01C    C01D    C01E    H01E    C01F    C01G
              C01H    H01H    C01I    C01J    H01F    H01G    H01I    C01K
              H01K    C01L    H01L    C01M    C01N    C01O    C01P    C01Q
              C01R    H01R    C01S    H01S    C01T    C01U    H01U
PLAT883_ALERT_1_G Absent Datum for _atom_sites_solution_primary ..     Please Do !
PLAT910_ALERT_3_G Missing FCF Reflection(s) Below Theta(Min)[Deg]=       4.10 Note
                0  0  1,
```

```
PLAT912_ALERT_4_G Missing # of FCF Reflections Above STh/L=  0.600        308 Note
PLAT913_ALERT_3_G Missing # of Very Strong Reflections in FCF ....          2 Note
                1  1  0,   2  2  2,
PLAT933_ALERT_2_G Number of HKL-OMIT Records in Embedded .res File         15 Note
                4 -2  7,   4 -9 12,   4 -6 18,   3-10 12,   4 -2  8,   4 -8 12,
               -3 11  0,   4-10 10,   4 -9 11,   3-11 11,  -2 12  0,   7 -1 18,
               -4 10  0,   4 -7 12,   2-11 13,
PLAT941_ALERT_3_G Average HKL Measurement Multiplicity ..........        2.7 Low
PLAT969_ALERT_5_G The 'Henn et al.' R-Factor-gap value ..........      2.919 Note
              Predicted wR2: Based on SigI**2  3.93 or SHELX Weight 10.68
PLAT978_ALERT_2_G Number C-C Bonds with Positive Residual Density.          0 Info
```

---

```
   0 ALERT level A = Most likely a serious problem - resolve or explain
   0 ALERT level B = A potentially serious problem, consider carefully
   6 ALERT level C = Check. Ensure it is not caused by an omission or oversight
  18 ALERT level G = General information/check it is not something unexpected

   2 ALERT type 1 CIF construction/syntax error, inconsistent or missing data
  11 ALERT type 2 Indicator that the structure model may be wrong or deficient
   7 ALERT type 3 Indicator that the structure quality may be low
   2 ALERT type 4 Improvement, methodology, query or suggestion
   2 ALERT type 5 Informative message, check
```

---

## Validation response form

Please find below a validation response form (VRF) that can be filled in and pasted into your CIF.

```
# start Validation Reply Form
_vrf_PLAT213_lcz078
;
PROBLEM: Atom F00U            has ADP max/min Ratio .....        3.1 prolat
RESPONSE: ...
;
_vrf_PLAT342_lcz078
;
PROBLEM: Low Bond Precision on  C-C Bonds ..............    0.00856 Ang.
RESPONSE: ...
;
_vrf_PLAT906_lcz078
;
PROBLEM: Large K Value in the Analysis of Variance ......      4.711 Check
RESPONSE: ...
;
_vrf_PLAT911_lcz078
;
PROBLEM: Missing FCF Refl Between Thmin & STh/L=    0.600         48 Report
RESPONSE: ...
;
_vrf_PLAT934_lcz078
;
PROBLEM: Number of (Iobs-Icalc)/Sigma(W) > 10 Outliers ..          1 Check
RESPONSE: ...
;
# end Validation Reply Form
```

---

It is advisable to attempt to resolve as many as possible of the alerts in all categories. Often the minor alerts point to easily fixed oversights, errors and omissions in your CIF or refinement strategy, so attention to these fine details can be worthwhile. It is up to the individual to critically assess their own results and, if necessary, seek expert advice.

---

**PLATON version of 17/09/2025; check.def file version of 17/09/2025**

---

# duplicate check

**No duplication found**

---

**Datablock lcz078** - ellipsoid plot

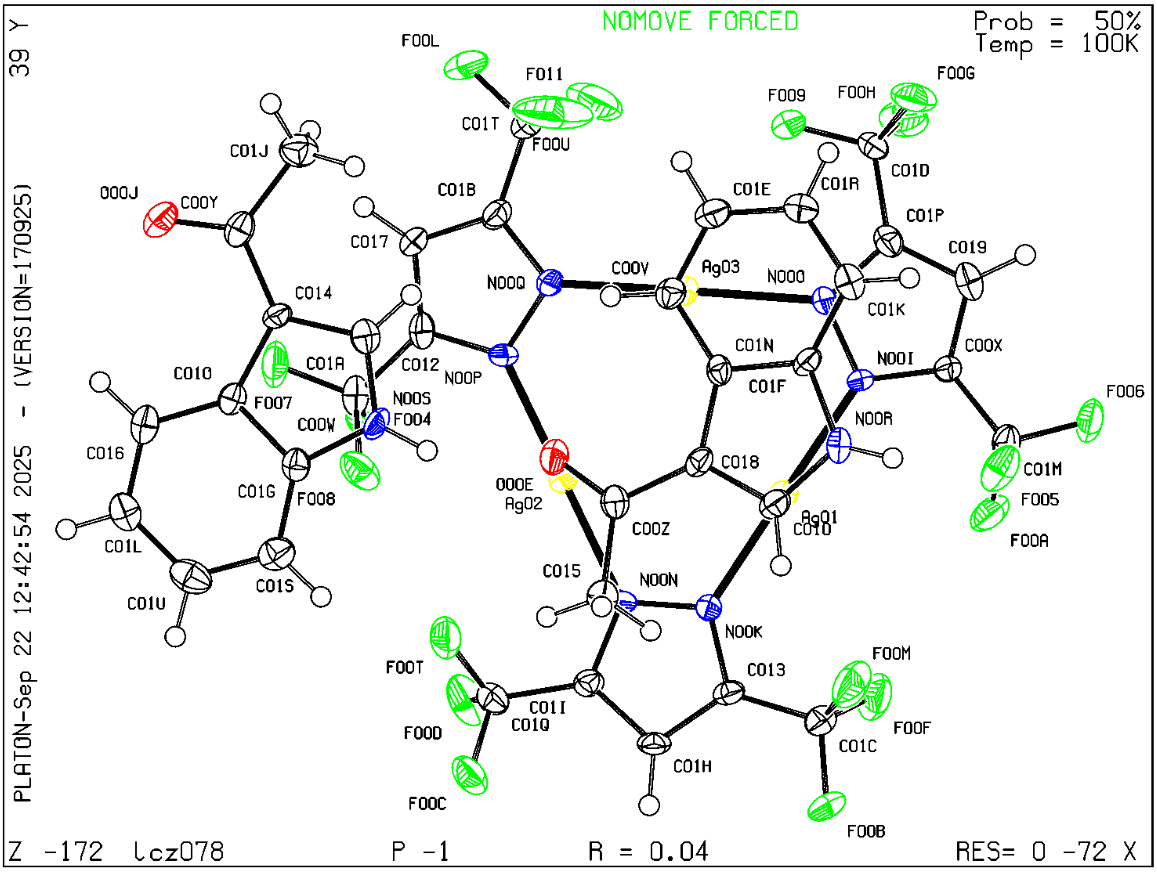

Supplement: Supplementary file 2 — Supporting File 2: anie72462‐sup‐0002‐DataFile.zip. [file ANIE-65-e1218503-s002.zip › supplementary_materials_crystallographic_data submit.zip!/cif_and_checkcif(1-114)/Ag3Pz3_78/Ag3Pz3_78_checkcif.pdf]
